# Multi-Fold Enhancement in Compressive Properties of Polystyrene Foam Using Pre-delaminated Stearate Functionalized Layer Double Hydroxides

**DOI:** 10.3390/polym12010008

**Published:** 2019-12-19

**Authors:** Emmanuel O. Ogunsona, Koffi L. Dagnon, Nandika Anne D’Souza

**Affiliations:** 1 Department of Materials Science and Engineering, University of North Texas, Denton, TX 76203-5310, USA; emmanuel.ogunsona@gmail.com (E.O.O.); kdagnon@gmail.com (K.L.D.); 2 Department of Mechanical and Energy Engineering, University of North Texas, Denton, TX 76203-5310, USA

**Keywords:** supercritical CO_2_, expanded polystyrene, layered double hydroxide, mechanical properties, compressive properties, nanocomposites, exfoliation, intercalation, in-situ polymerization

## Abstract

Developing an environmentally benign styrene foam is a critical environmental need. Supercritical CO_2_ use in foams has proven to be a valuable path. Adding fillers to increase bubble nucleation has been pursued concurrently. A prominent filler used is high surface area fillers, such as smectic clays. However, all studies to date show a limit of 152% in compressive moduli and 260% in the compressive stress. The values, even with such gains, limit structural application. A seminal work in 1987 by Suh and Cotton proved that carbonyl linkages in calcium carbonates and CO_2_ interact and impact nucleation efficiency and performance in supercritical CO_2_ foams. In this paper, a high surface area clay (layer double hydroxides) which begins in an exfoliated state, then functionalized with a long chain alkyl carboxylate (stearic acid) is synthesized. The result is a remarkable multi-fold improvement to the compressive properties in comparison to polystyrene (PS); a 268% and 512% increase in compressive modulus and strength, respectively. Using a pre-delaminated approach, the higher surface area was achieved in the clays. The presence of the stearate improved the interactions between the clay galleries and PS through hydrophobic-hydrophobic interactions. The glass transition temperature of the nanocomposites was observed to shift to higher values after foaming. The results point to a new path to increase performance using a pre-delaminated clay with functional groups for environmentally benign foams.

## 1. Introduction

Polystyrene (PS) is one of the major commodity plastics widely used and commercially produced [1], with an annual production of 19.6 million tons, as of 2015, and has continued to grow ever since [2]. It is used for applications in various sectors, such as building and piping, automotive, electronics and electrical equipment, furniture and food packaging, just to mention a few [2,3]. However, due to the ever-growing demand for petroleum products, leading to depleting petroleum resources, the need to limit the use of this polymer is essential to aid in curbing the aforementioned problems. Environmental pollution from plastic waste is also of great concern, as a third of PS produced is used as packaging material, which eventually makes up one of the major fractions of municipal solid wastes [4]. One of the innovative ways of reducing the consumption of petrol-based polymers, such as polystyrene is by increasing its volume to weight ratio through the introduction of voids by means of the foaming process [5].

Physical foaming techniques have evolved over the years, and the especially the expansion of polystyrene using foaming agents, such as water and supercritical carbon dioxide (scCO_2_) has led world-wide research on enhancing its foamability and subsequently, its thermal and mechanical properties. Currently, the low mechanical properties (especially compressive properties) of foamed polystyrene has limited its use to nonstructural applications, such as packaging and non-load bearing building insulation. It was found that by introducing impurities or fillers into a polymer, cell nucleation and growth around that filler can be induced, due to the creation of free volume at the interface between the filler and polymer [6,7]. By doing so, well-dispersed nanofillers can bring about simultaneous cell nucleation sites and homogeneous cell growth, leading to smaller cell sizes and increased cell densities [8]. Chen et al. were able to disperse carbon black (CB) in poly (butylene succinate) to produce uniform cell distribution even at a high loading of 10 wt.%, leading to 108% increase in compressive strength by using a kinetic batch mixer [9]. Ju et al. introduced a pre-foaming enhancing process by orienting the crystal structure of poly (L-lactic acid) (PLLA) through hot plate compression of PLLA blends with poly (ethylene glycol). The result was an interconnecting open-cell structure with ca. 90% porosity, which was well suited as scaffolds for tissue cell growth [10]. A scale-up and low cost foamed polystyrene micro-graphite and multiwalled nanotube composite foams were fabricated using high shear extrusion compounding and then foaming. The cell foam structure was shown to be significantly affected by the type and structure of the filler used [11]. Therefore, filler dispersion through pre-foaming processes such as twin screw shear mixing [12] and filler type can significantly affect the cell nucleation, leading to favorable foam structures. Likewise, if the filler is well embedded in the cell walls, this can also enhance the mechanical properties by multiple folds.

Fei et al. studied the supercritical foaming of PS using lignin, multiwalled carbon nanotubes (MWCNT) and micro-graphite (mGr) as heterogeneous nucleating agents [13]. They revealed that the cell sizes of the foams reduced with increasing filler loading. It was also observed that the MWCNT exhibited the most reduction in cell size and density, as well as the best cell homogeneity, due to its size in the nano range. However, minimal increases in the specific compressive strengths were observed for foams containing MWCNT and mGr when considering the standard deviation. Foams containing lignin exhibited the opposite results; the specific strength reduced with increasing lignin loading. A comparison of PS foam properties in the presence of unmodified and modified montmorillonite layered silicate (MLS) with a cationic surfactant (2-methacryloyloxyethylhexadecyldimethyl ammonium bromide) and talc was made [14]. Results showed that the modified clay was exfoliated in the PS, resulting in improved dispersion and subsequently increased cell density and homogeneity in comparison to the unmodified clay and talc. However, the increase in modulus observed was subtle in relation to the neat foamed PS. This outcome mirrors our previous attempt at improving the compressive performance of polystyrene foam. Previously, an alkyl ammonium modified MLS smectite clay was investigated [15]. When MLS was introduced into the PS, the chains interacted with the alkyl ammonium cation. Compressive yield strength and modulus went from 0.4 to 0.77 MPa (92.5% increase) and from 11.25 to 28.35 MPa (152% increase), respectively, due to good adhesion between the MLS and PS. Other researchers investigated montmorillonite and polystyrene supercritical CO_2_ and reported enhanced cell density with no concurrent mechanical properties reported [16,17]. 

In this paper, the benefits offered by platelet-shaped fillers together with a fundamental exploration espoused a few decades earlier in developing the technologies for CO_2_ foams by Colton and Suh [18] was harnessed. In 1987 they investigated the role of additives on nucleation. Using zinc stearate, their work indicated that carboxylate enhanced interaction with CO_2_ and resulted in higher cell nucleation efficiency. In this paper, a new pathway is explored through the coupling of the carboxylate linkage to the platelet geometry afforded by smectite clay and investigation on the impact on the cell density, as well as on mechanical properties of PS. Furthermore, increases to the polymer-filler interfacial area is achieved by using a pre-delaminated clay. Through the novel pathway of creating pre-delaminated stable structures of uniform shapes to increase surface area [15], layered double hydroxide (LDH) in our exploration. 

LDH, a form of synthetic anion exchange smectic clays, are based on mixed metal hydroxides (also called hydrotalcites) used in a variety of technological applications, such as separation, ion-exchanges and absorbance [19]. The mixed metal hydroxides have an overall formula of [M^II^_1−x_M^III^_x_(OH)_2x_]^x+^(A^n^^−^)_x/n_.mH_2_O, where M^II^, M^III^ and A^n^^−^ represent a divalent metal, a trivalent metal and an anion, respectively [20]. In the brucite-like structure of LDH, a fraction of the divalent metal is replaced with a trivalent metal. The presence of the trivalent metals imparts a positive charge to the layers; hence, the electroneutrality of the LDH is maintained through the introduction of anions intercalated between these layers [8,9,10]. The M^II^ divalent and M^III^ trivalent metals used were magnesium and aluminum, respectively, while nitrate was used as the anion because exchange with stearate is possible [21]. 

## 2. Materials and Methods

### 2.1. Materials

Aluminum nitrate nonahydrate, n-hexadecane and sodium hydroxide (NaOH) solution were obtained from Alfa Aesar Chemicals by Thermo Fischer Scientific, Tewksbury, MA, USA. *Zinc* nitrate hexahydrate, toluene, p-tert-butylcatechol, benzoyl peroxide, n-hexane and stearic acid, 95% purity were purchased from Sigma-Aldrich, MA, USA. Styrene monomer was purchased from Fisher Scientific, MA, USA. All chemicals were used as-is without any further modification.

### 2.2. Synthesis of Stearate Functionalized Layered Double Hydroxide

Nitrate functionalized layered double hydroxide (Zn-Al NO_3_ LDH) was synthesized following similar procedure as described by Koffi et al. [22]. Using the Zn-Al NO_3_ LDH, stearate functionalized LDH was then obtained through the following procedure. 499 g of *zinc* nitrate hexahydrate (8.40 mmol) and 1.047 g of aluminum nitrate nonahydrate (2.79 mmol) were solubilized in deionized water (50 mL). 0.88 mL of 50% NaOH solution concentration (16.80 mmol) was added while stirring under nitrogen gas flow. The mixture was then matured for 24 h under constant stirring and a nominal external oil bath temperature of 100 °C. The reaction was stopped by cooling the mixture and then centrifuged. The precipitate was then washed with several times to obtain ZnAl-LDH-NO_3_ precipitate. A stearic acid solution was prepared by an exchange reaction was performed by adding a solution of stearic acid prepared by neutralizing a dispersion of 0.794 g stearic acid (2.79 mmol) in deionized water (50 mL) with a solution of 0.10 mL of 50% NaOH. This mixture was allowed to agitate by stirring for 60 min, centrifuged and then washed multiple times using deionized water. The obtained precipitate was vacuum dried over molecular sieves and referred to as as-prepared ZnAl-LDH-stearate. 

The as-prepared ZnAl-LDH-stearate was aged in 50 mL n-hexadecane overnight in an oil bath at 120 °C under vigorous mixing. After stopping the mixing and cooling, the delaminated-LDH sank to the flask bottom. The delaminated-LDH was separated by centrifugation and washed with n-hexane. It was then collected, dried overnight and stored in a desiccator containing molecular sieves until further use. This is referred to as as-exfoliated ZnAl-LDH-stearate. 

Elemental analysis of the LDH-stearate shows that, compared to the theoretical value, the stearate is slightly over-exchanged (Table 1).

### 2.3. Preparation of PS and PS-LDH Nanocomposites

**Scheme 1:**
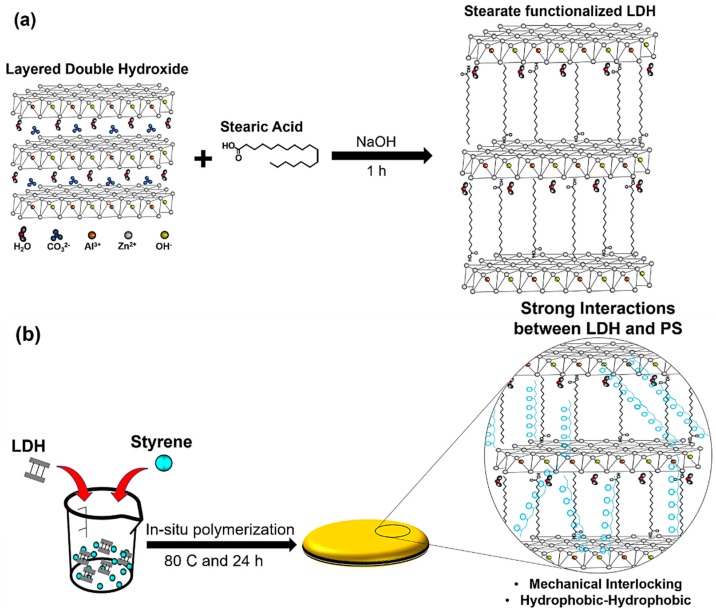
Illustration showing (**a**) the functionalization of layered double hydroxide (LDH) using stearic acid resulting in an increase of the interlayer d-spacing of the LDH and hydrophobicity, and (**b**) in-situ polymerization of polystyrene-layered double hydroxide (LDH1, LDH3 and LDH5) nanocomposites showing strong interactions through hydrophobic-hydrophobic and mechanical interlocking.

Polystyrene (PS) was prepared by the solution polymerization method. The delaminated LDH (1, 3 and 5 wt.%) was added to 50 mL toluene in a two-necked flask in order to help facilitate dispersion of LDH in the monomer. The solution was mixed for 60 min at room temperature. Appropriate amounts of styrene with 15 ppm p-tert-butylcatechol stabilizer and benzoyl peroxide (0.2 wt.%) initiator were added to the stirring solution in the flask. The ratio of the toluene to monomer used was 1:4. The mixture was further stirred at room temperature for about 30 min until the delaminated LDH monomer solution appeared to be visually fully dispersed, and then heated to 80 °C for about 24 h to initial and complete polymerization. These as-prepared samples are denoted as PS, LDH1, LDH3 and LDH5 corresponding to 0, 1, 3 and 5 wt.% respectively, which are the concentrations of LDH used. 10 g disk samples were made by pressing the prepared nanocomposites in a mold under 40 MPa of pressure and at 80 °C for characterization. In Scheme 1a, the illustration demonstrates the functionalization of LDH with stearic acid to produce stearate functionalized LDH as described above, with increased d-spacing for improving dispersion when utilized in polymer composite applications. In Scheme 1b, a demonstration of the in-situ polymerization of polystyrene and LDH results in well-dispersed LDH in the polystyrene, subsequently leading to improved interactions between them through hydrophobic-hydrophobic interactions and mechanical interlocking.

### 2.4. Supercritical Carbon Dioxide Foaming of PS and Nanocomposites

A Fritsch pelletizer (Fritsch, Idar-Oberstein, Germany) was used to pelletize PS and its nanocomposites. The pelletized samples were dried for a day at 30 °C, and then compression molded into sheets with dimensions of 2.45 × 40 × 40 mm using a Carver hydraulic hot press. The press was operated at a pressure and temperature of 6 tons and 185 °C, respectively, for 15 min. The samples were foamed in accordance with optimized foaming procedures by Strauss et al. [23], where samples were placed in a pressure reactor, sealed and filled with CO_2_. The reactor was then heated to 37 °C and 1175 psi, which are the conditions for the supercritical state of CO_2_. The samples were allowed to soak for 15 min at this state to allow ScCO_2_ penetration and saturation. After this, the reactor was suddenly depressurized, causing thermodynamic instability of the ScCO_2_ and bubble formation in the sample as the CO_2_ gas is removed.

### 2.5. Thermal Analysis of PS and PS-LDH Nanocomposites

The effect of LDH and foaming on the thermal transitions of the samples were determined using a Perkin Elmer DSC-6 differential scanning calorimeter (DSC) (Perkin Elmer, Cincinnati, OH, USA). Approximately 5 to 10 mg of sample was used and evenly spread in the non-volatile aluminum pan and sealed. The samples were kept for 5 min at an isothermal temperature at 5 °C and then heated to 200 °C at a heating rate of 10 °C/min. The samples were then held at 200 °C for 5 min and then cooled down to 5 °C at a rate of 10 °C per minute. This cycle was repeated twice.

### 2.6. Characterization and Morphological Analysis of Foamed Nanocomposites

The densities of the polymer (*ρ*_f_) and foam (*ρ*_m_), were determined according to ASTM standard D1505-18 and D1622-14 methods. As suggested by Kumar and Weller [24], the relative foam density (*ρ*_r_) was determined. The foamed sample cell structures were characterized using a Quanta 200 environmental scanning electron microscope (ESEM) (Thermo Fischer Scientific, OR, USA) by scanning the surface of cryo-fractured samples to maximize the exposure of the true diameter of the cells as a fracture propagates along its weakest axis. These surfaces were then gold-coated to prevent charging using a sputter coater.

From the micrographs obtained, the average cell diameters (*D*), cell density (*N_o_*), volume density (*N_f_*) and void fraction (*V_f_*) using methods suggested again by Kumar and Weller [24] were determined. A detailed explanation of the process is described by Ogunsona et al. [15].

Transmission electron microscopy (Philips EM 420 TEM) (Philips, Mahwah, NJ, USA) was used to determine the LDH dispersion within the PS matrix as an auxiliary to the XRD (Rigaku model D/Max Ultima III X-ray diffractometer (Woodlands, TX, USA). SEM and cell characterization analysis of the composites. The images were captured using an EM-420 model analytical TEM instrument manufactured by Philip at an accelerating voltage of 120 kV. Samples were prepared by embedding the nanocomposites into epoxy resin and then slicing them into thin sheets of about 90 nm using a microtome (RMC MT 6000).

### 2.7. Mechanical Properties

Compression tests were performed on the foamed PS and PS-LDH nanocomposite samples according to ASTM standard D1621-16, using a hydraulic MTS machine (MTS Systems Corporation, Minneapolis, KS, USA) at a crosshead rate of 0.1 in/min and under room temperature. Prior to testing, the foamed samples were cut into circular shapes with a thickness and cross-sectional area of 2.54 cm and 25.8 cm^2^, respectively, then conditioned at room temperature and 50% relative humidity.

## 3. Results and Discussions

### 3.1. X-Ray Diffraction Analysis

**Figure 1:**
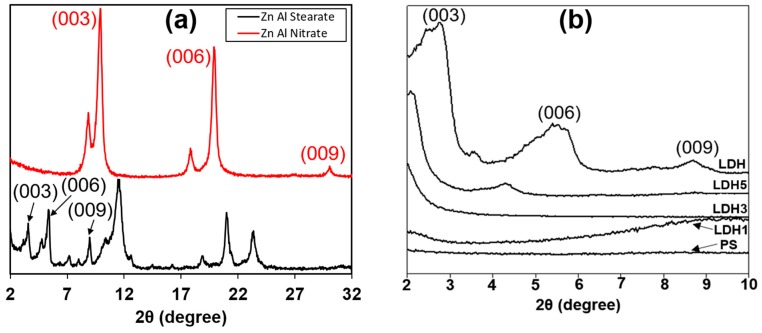
X-ray diffraction patterns of (**a**) Zn Al stearate and Zn Al Nitrate showed the changes in the d-spacing, due to stearate penetration into the galleries and (**b**) the unfoamed polystyrene (PS) and PS-LDH nanocomposites demonstrating the interactions between the PS and stearate-LDH leading to intercalation and exfoliation of the layers.

**Scheme 2:**
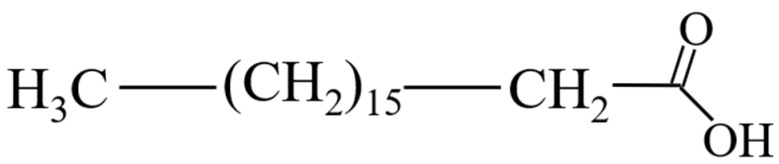
Chemical structure of stearic acid.

**Scheme 3:**
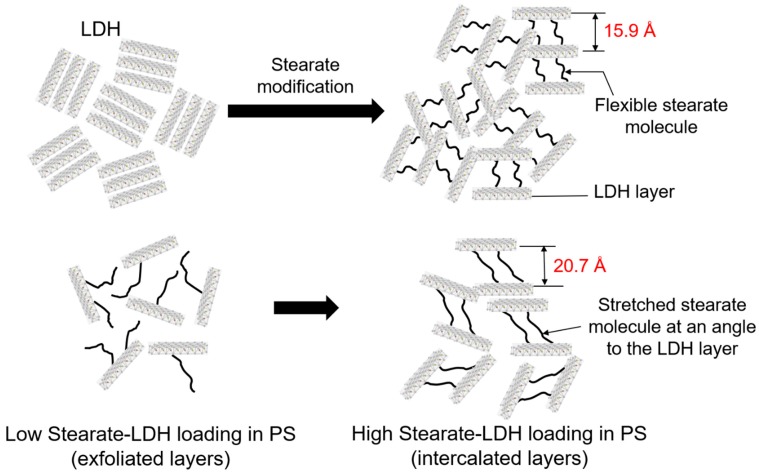
An illustration of the LDH interlayer separation, due to modification with stearic acid and the effect of weight concentration in polystyrene thereafter.

In Figure 1, x-ray diffraction was used to analyze the Zn Al nitrate and stearate after modification and the dispersion of the modified LDH in the nanocomposite samples. A comparison of the XRD patterns of both Zn Al nitrate and stearate, as shown in Figure 1a, reflect changes of the diffraction pattern to lower angles. A shift of the (003) peak to a 2θ of 3.56° for of the Zn Al stearate from 9.98° for the Zn Al nitrate is observed which is associated with the interlayer distances [22], resulting from the replacement of the nitrate ions with those of the stearate, hence, causing an increase in the interlayer distance. The change in interlayer distance (d spacing) was calculated to be ca. 15.94 Å, which is significantly lower than the expected length of the stearic acid of ca. 24.2 Å as determined by Lane et al. [25]. It is known that the lack of carbon double bonds on the stearic acid (Scheme 2) leads to the flexibility of its hydrocarbon chain and can be stretched out into a long zig zag [26]. Therefore, this discrepancy could be due to the flexible alkyl chains of the stearic acid bending and folding, leading to shorter distances between the interlayers as demonstrated in Scheme 3. 

The XRD patterns of the nanocomposites are presented in Figure 1b. A complete disappearance of the (006) and (009) peaks in LDH1 and LDH3 is observed, which indicates complete exfoliation of the LDH nanoparticles in the matrix polymer (PS). This also indicates the well and finely dispersed phases within the polymer matrix. Hence, it is expected that more homogenous cell size, distribution and density will be achieved for these nanocomposites when foamed, since cell nucleation is increased in the presence of particles or impurities. This is because CO_2_ tends to accumulate at the interfaces between particles and polymer matrices.

The presence of a residual peak in LDH5 remains. However, the decrease in peak height of the (003) indicates that the result could be a result of packing efficiency. As the LDH concentration increased, the probability of two LDH having alignment to contribute to an XRD peak increased. The (003) peak shifts to a 2θ of 4.27°, representing an increase to 20.7 Å of the d-spacing of the intercalated layers from the original d spacing of the stearate modified LDH of 15.94 Å. As determined earlier, the stearic acid length of 15.94 Å suggests that during in-situ polymerization of the nanocomposites at LDH loading of 1 and 3 wt.%, the interlayers of the stearate modified LDH are further separated by the PS molecules, plausibly due to hydrophobic-hydrophobic interactions occurring between the stearic acid and PS molecules. Hence, resulting in exfoliation of the LDH with an interlayer distance of 27.5 Å; 3.3 Å longer than the length of the stearic acid. Strauss and D’Souza found that the d-spacing of 1 wt.% of montmorillonite (MLS) in PS after foaming increased from 3 to 3.4 nm and suggested that the laminates might have been swollen from the saturation of the foaming agent [27]. It is also likely that the laminates are further pushed apart during the destabilization of the foaming agent in the PS. 

At higher loadings of LDH (LDH5), the interlayer is reduced to 20.7 Å, which shorter than the length of the stearic acid by 3.5 Å. This change in interlayer distances can ostensibly be attributed to an increase in layer-layer contact, leading to intercalation as observed in TEM images. However, an increase in the interlayer distance from 8.86 Å of the Zn Al nitrate to at least 20.7 Å is tremendously remarkable. Therefore, a structure containing the stearate alkyl chains is expected to favor the dispersion of the LDH in the styrene monomer during in situ polymerization, leading to increased mechanical performance. An illustration showing the changes to the interlayer distances of the stearate modified LDH with respect to the effect of its surroundings is shown in Scheme 3.

### 3.2. Effect of LDH and Foaming on the Thermal Properties of PS and PS-LDH Nanocomposites

**Figure 2:**
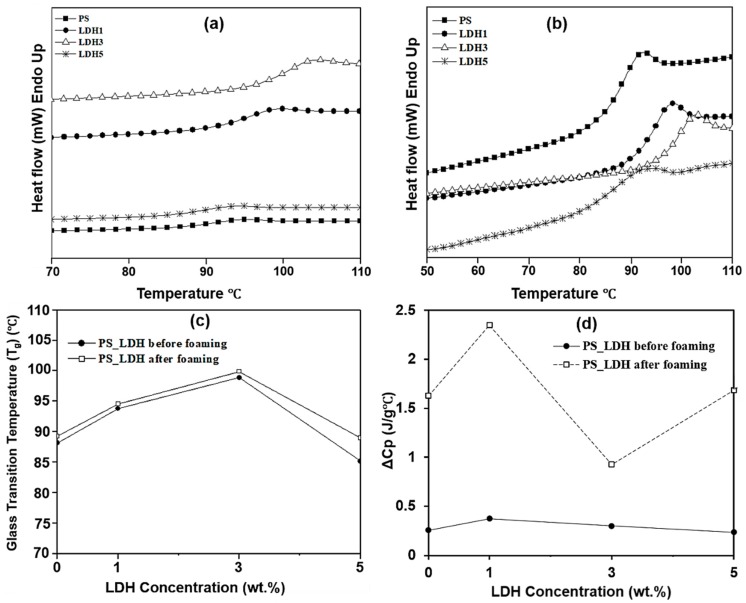
First heating curves from differential scanning calorimeter (DSC) analysis for (**a**) unfoamed and (**b**) foamed PS and PS_LDH nanocomposites and the effect of ScCO_2_ on the (**c**) glass transition temperature and (**d**) change in enthalpy with respect to LDH concentration.

The DSC results of PS and its nanocomposites for both the unfoamed and foamed samples are shown in Figure 2a,b, respectively. It can be observed that the incorporation of 1 and 3 wt.% of LDH into PS causes glass transition temperature increases successively. At 5 wt.% of LDH it is observed to reduce, similar to the that of the neat PS. This observation can be attributed to the LDH acting to restrict localized chain vibration of the PS at elevated temperature, therefore, requiring higher temperatures to achieve this. Increasing the weight fraction (3 wt.%) begins to obstruct and reduce the formation and size of crystallites, due to more LDH-LDH interactions. At higher LDH loading (5 wt.%), there is reduced dispersion and intercalation occurring; hence, the effect is reduced and similar to the glass transition temperature (*T*_g_) of the neat PS. 

Figure 2b shows how the foaming process affects the *T*_g_ of the composites. It can be observed that the *T*_g_ of the foamed samples follow similar trends as those of the unfoamed samples. However, there is a slight increase after foaming for all samples, suggesting further restriction of the chain from vibrating. As explained in our previous work using MLS [15], it is suggested that plasticization effect of the CO_2_ cause partial mobility of the PS chains, causing them to orientate in the direction of the escaping gas during the thermodynamic destabilization process (depressurization). Likewise, the LDH is capable of some level of alignment, as well as a result of the chain realignment. This is similar to the realignment of some chains in polymers in an injection molded part [28] or a comb through tangled hair; the CO_2_ acts as the teeth of the comb in the chain realignment process. A plot of the unfoamed and foamed *T*_g_ against LDH concentration is shown in Figure 2c, with the LDH5 exhibiting the most pronounced difference. This occurs because the intercalated LDH is better dispersed after foaming, thereby having a more restrictive effect on the chain vibration of the PS.

In Figure 2d, the change in specific heat (*ΔCp*) is plotted against the LDH concentration before and after foaming. It can be observed that the total energy required to break the intermolecular bond between the PS chain remains mostly the same for the unfoamed PS and nanocomposites. However, this is significantly increased after foaming of the PS and nanocomposites, with LDH1 showing the highest increase in *ΔCp*. This is reflective of the increased glassy state caused in the polymer from exposure to supercritical CO_2_. The data obtained from the differential scanning calorimetric analysis is shown in Table 2.

### 3.3. Characterization of PS and PS-LDH Nanocomposite Foam Properties

**Figure 3:**
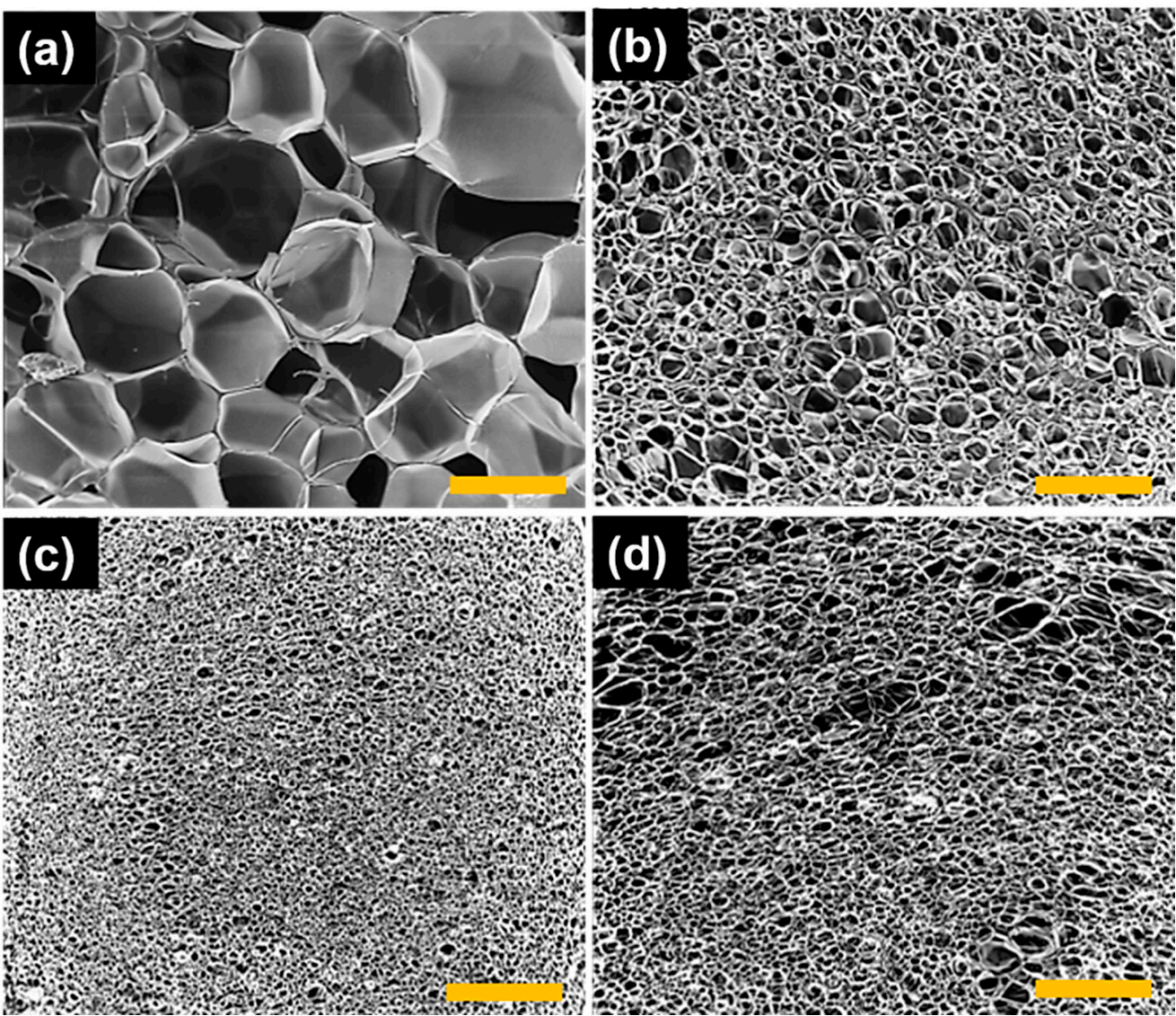
SEM micrographs of (**a**) PS, (**b**) LDH1, (**c**) LDH3 and (**d**) LDH5 expanded at 80 °C and 1175 psi using ScCO_2_. *(The scale bar in the figure represents 1 mm)*.

The micrographs and data analyzed from the PS and nanocomposites foams are shown in Figure 3 and Table 3, respectively. It can be seen that the cell size progressively reduces with the addition of 1 and 3 wt.% of LDH and then increases at 5% LDH loading. In our previous work, a similar trend was found in PS nanocomposite foams reinforced with montmorillonite layered silicate (MLS) [15]. It is expected that the cell density would increase with the subsequent addition of LDH up to 5 wt.% loading since cell size known to be inversely related to the cell density. This increase in cell size and reduction in cell density in LDH5 can be attributed to increased interaction and contact of individual LDH particles with each other, which increases the nucleation site area. The greater the agglomerates, the bigger the nucleation site and subsequently the cell size. This results in larger individual cell formation. Hence, it can be concluded that the large cells observed in LDH5 are nucleation sites where intercalated LDH are found. 

From Table 3, it can be seen that the cell density increases with increasing LDH. This can also be seen by studying the micrograph of the foamed samples. All the foamed samples except neat PS exhibit open cell structures (individual cells with no connection to others). This property of expanded PS is where its application as a thermal insulator is derived from. The air pockets in the cells help reduce heat loss. A similar trend in the reduction of cell size and increase in cell density was observed in foamed polycaprolactone, and mater-bi blends as mater-bi concentration were increased [29]. Mater-bi is a thermoplastic starch, and therefore, contains starch particles which can be present in the nano-size. The presence of starch was attributed to the formation of nucleating sites, and therefore, increased cell density and reduced cell size. The stearate functionalized LDH followed showed a significantly higher nucleation efficiency. The cell size was close to half to the third of that reported in our PS with MLS work [11].

**Figure 4:**
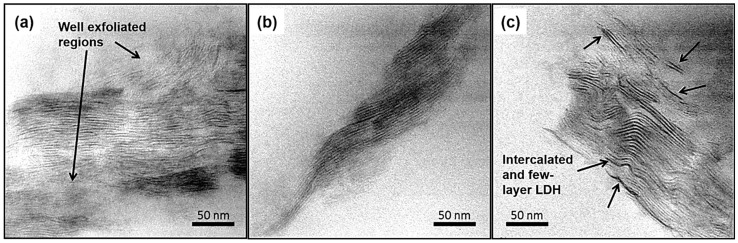
TEM micrographs of PS/LDH nanocomposites: (**a**) LDH1 (**b**) LDH3 and (**c**) LDH5.

TEM was used to observe the dispersion of the LDH in PS. It can be seen that there is good dispersion (exfoliation) in LDH1 and LDH3, but some agglomeration in LDH5 (Figure 4). The presence of thicker lines in LDH5 represent layers of platelets which are not exfoliated, as pointed out by the arrows. This result is in correlation with those from the XRD and supports the results obtained from foam morphology and characterization. As stated earlier, the high loading of LDH in LDH5 resulted in incomplete exfoliation or more intercalated layers, subsequently resulting in reduced wetting by the matrix, larger voids at interfaces, and increased and larger nucleation sites. As a result of the larger nucleating sites, there was an increased cell size, reduced foam density, and exfoliation within the matrix in LDH5. 

### 3.4. Mechanical Properties—Compression Test

**Figure 5:**
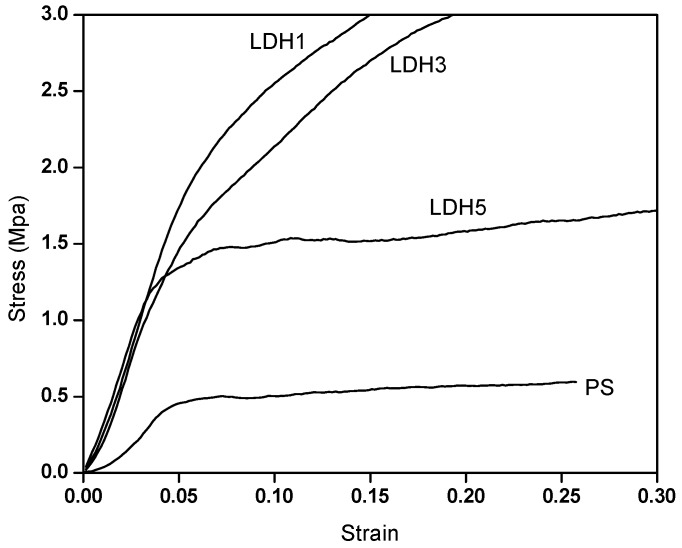
Stress-strain curves from the compressive test of PS and PS_LDH nanocomposites foams.

The stress-strain plot of the compression test of the foamed nanocomposites is shown in Figure 5. It can clearly be observed that the addition of LDH to PS significantly increases the compressive properties. Relative to our previous work using MLS [15] at the same filler concentration, the modulus was found to be 19.84 MPa (MLS1), whereas LDH1, in this case, ne with fur was at 41.44 MPa. The compressive strength of the LDH1 is 300% that of the MLS1. The improvement observed in LDH1 can be attributed to the stearate functionalization of the LDH. Strong mechanical interlocking between the exfoliated LDH stemming from the attached stearate molecule improves the interfacial adhesion with the PS. Likewise, well-dispersed LDH in the PS can be attributed to increased hydrophobic-hydrophobic interactions between the stearate on the LDH and the PS, as seen from the XRD patterns of the samples. All of these contribute to improvements in mechanical performance. 

The compressive properties are observed to decline with the further addition of LDH, especially for LDH5. It is well known that nanofillers begin to negatively affect mechanical properties as their concentrations increase, even at low loadings of 3 and 5 wt.%. This is due to the high surface area and aspect ratio, which results in aggregates and agglomeration, eventually leading to poor interactions with the matrix. This is clearly observed from the XRD and TEM results; at low loadings (LDH1 and 3) the LDH is exfoliated, and observed to only be intercalated at higher loadings (LDH5).

The compressive properties (yield stress, Young’s modulus, and compressive strength) resulting from the test are listed in Table 4. The improved compressive properties are due to the stiffening of the samples by LDH, which interferes with chain mobility of the matrix, while providing good stress transfer to the particles from the matrix. Overall, the compressive properties of the PS are enhanced by the addition of LDH nanoparticles, and the optimum concentration of LDH is at 1%.

### 3.5. Theoretical Modeling of the Relative Compressive Moduli of PS and PS Composite Foams

The cell structures of the foamed samples were studied by employing theoretical models which predict the relative compressive modulus by making assumptions regarding the type of cell structure. Three models; Gibson and Ashby [30], Chen (Closed-cell constitutive) [31] and Naguib [32] were applied. The Gibson and Ashby model describe the relationship between the relative modulus (*E_r_*) and relative density (*ρ_r_*) of the foams. It assumes a closed cell structure with the volume fraction of polymer required to construct the edges of the cells to be ɸ = 0.9. The relationship between the relative modulus and the relative density was calculated using Equation (1):

(1) $$E_{r}={ɸ}^{2}\cdot \rho_{r}{}^{2}+\left(1-ɸ\right)\rho_{r}.$$

The Chen model, which also describes the relationship between the relative modulus and relative density assumes that the cell structure is closed and takes into account the cell edge thickness and lengths. Thereby, predicting more accurately the experimental data by eliminating the assumption that ɸ = 0.9, which does not always apply in all cases. The model was computed using Equation (2):

(2) $$E_{r}=\;\left(\frac{3A_{r}^{2}-\;2A_{r}^{3}}{1-\;{\left(1-\;A_{r}\right)}^{3}}\right)A_{r}^{4}-\;\beta \left(1-\;\frac{3A_{r}^{2}-\;2A_{r}^{3}}{1-\;{\left(1-\;A_{r}\right)}^{3}}\right)\frac{A_{r}}{2},$$

(3) Ar=1− (1− ρr)13,

where *A_r_* is the ratio of the edge thickness over the edge length and obtained using Equation (3). *β* is a constant of proportionality and calculated by substituting the experimental values of *Er* and *ρ_r_* for neat PS in Equations (2) and (3). The value for *β* obtained and used was 0.313.

The Naguib model, similar to the Chen model assumes an open cell structure; hence, the account for cell faces is neglected. The model was computed using Equation (4):

(4) $$E_{r}=\;C_{0}{\left(0.14159+0.7082\rho_{r}\right)}^{4},$$

where *C*_0_ is a constant determined by fitting to the experimental data. The experimental relative modulus was calculated by dividing the compressive moduli of the PS foams by the compressive modulus of PS.

**Figure 6:**
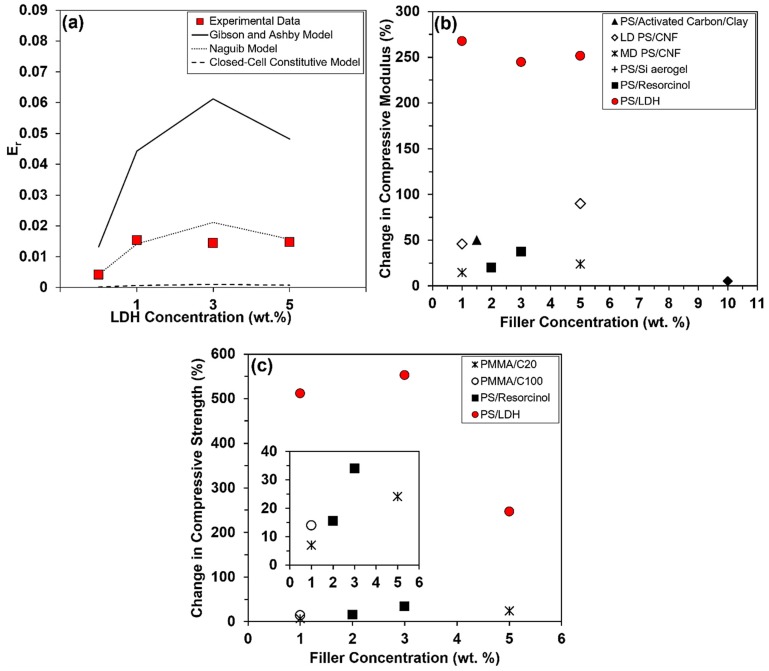
Graphical representation comparing (**a**) relative modulus (E_r_) models to experimentally determined data as a function of the LDH concentration and benchmarking the percentage increase in (**b**) compressive modulus and (**c**) compressive strength of experimental data against PS composites from in the literature, due to incorporation of fillers [31,35,36,37].

It can be observed from Figure 6a that the Gibson and Ashby model does not fit well with the experimental data beyond the neat PS foam, even though it has been applied successfully to foams with low relative densities foams [33,34]. This is because the model assumes a closed cell structure with an effective relative foam density of <0.2 and considers the longitudinal dimension to be significantly greater than the latitudinal dimension. Hence, assuming the shape of the cell structure to be rod-like. However, samples containing LDH in the current study do not meet these criteria; they have open cell structures and relative foam densities closer to the boundaries for the effective relative foam densities of 0.2. This results in predictions that do not fit well. Similarly, the assumption of the volume fraction of polymer required to construct cell edges is a constant of 0.9 may not always apply, especially for increasing foam densities.

The Chen or closed-cell constitutive model also exhibits a non-fit, which is the extreme opposite of the Gibson and Ashby model. This model, like the Gibson and Ashby model, also assumes a closed cell structure, but assumes that the cells are cubic with equal edges and face. Hence, better fitting the experimental data that the Gibson and Ashby model. However, due to the assumption that the cells are closed, a misfit is observed. When the Naguib model is fitted to the experimental data, a very close similarity is observed. In this model, the assumptions are similar to those of the Chen model except that the cells are considered to be open cell. This close-fit to that of the experimental data suggest that foam samples containing LDH have open cell structures as suggested and observed earlier through the morphological analysis. Literature suggests that this model fits well for polymer foams with relative densities ranging from 0.2 to 0.5 [8].

### 3.6. Benchmarking the Compressive Properties of PS Nanocomposites Against Literature

The results from the compressive properties (strength and modulus) were benchmarked against those extracted from the literature to showcase the significance and potential of stearate functionalized LDH on the mechanical properties of foamed PS. All fabricated PS composites foams from the literature were produced in identical processes as their counterpart PS. Figure 6b shows the percentage change in modulus of the experimental data (PS/LDH) plotted against PS composites foams from the literature, fabricated using different filler types and at the same filler concentration. It can be observed that the percentage change in modulus for the experimental data at all LDH concentrations supersede those found in the literature by at least 2.5 times. This shows the effectiveness of stearate functionalized LDH in the foaming and reinforcement of PS in comparison to other nanomaterials, such as carbon nanofibers and nanoclays, which have excellent load bearing and polymer chain restricting capabilities. The percentage change in compressive strength of the experimental data is shown in Figure 6c with even more remarkable superior enhancement in comparison to those in the literature. In this case, at least a 7-fold increase is observed.

This remarkable multi-fold enhancements in comparison to literature can plausibly be attributed to increasing in d-spacing of the LDH by the stearate, allowing for the PS chains to mechanically interlock during in situ polymerization and the hydrophobic-hydrophobic interaction between the PS chains and the stearate in the LDH, as seen in the illustration in Scheme 1.

## 4. Conclusions

The approach of stearate functionalization to improve nucleation in supercritical CO_2_ foamed polymer composites espoused by Cotton and Suh in 1987 proved effective in conjunction with clay filled polymers. A synthetically prepared layered double hydroxide was functionalized with stearate and introduced into polystyrene in weight fractions of 1, 3 and 5% w/w. The LDH was successfully exfoliated in the in-situ synthesized polystyrene at 1% and 3% and had an intercalated dispersion at 5%. The compression properties of the resulting foams were significantly enhanced by multi-fold of the modulus and strength. Most significantly, relative to polystyrene modified with MLS investigated previously; and the foams showed a 100% improvement. This was linked to enhanced dispersion through stearate functionalization and nucleation efficiency of the stearate-CO_2_ supporting the hypothesis. The cell size decreased, and cell density increased in the LDH-stearate functionalized foams. The results point to a new approach for gaining significant strength improvements in foamed polystyrene.

Martinez et al. investigated a commercial Mg, Al carbonate layered double hydroxide treated with dodecylsulfate (DDS) to increase the d_003_ interlayer spacing to ~3 Angstroms [38]. The resulting PS-LDH nanocomposite did not show a shift in the 2-theta peak, nor was there any shift in the glass transition temperature between foamed and unfoamed polymer. The storage modulus of the PS and PS-LDH foams remained unchanged in the glassy region, and a loss was observed in the post-*T*_g_ region. However, the LDH-DDS impacted the number of cells per cubic centimeter significantly, indicating an effect on cell nucleation. Though, this did not translate to increased mechanical performance. 

## Figures and Tables

**Table 1:** Elemental analysis of synthesized layer double hydroxide (LDH)-stearate.

LDH Sample	Zn (%)	Al (%)	C (%)	H (%)	Zn/Al ratio	C/Al ratio	Residue (%)
As-prepared Zn Al-stearate	20.2	4.19	42.4	8.43	1.99	22.7	28.0
Zn_2_Al(OH)_6_(stearate)1.1.2H_2_O	22.6	4.66	41.0	8.37	2.00	19.8	38.1

**Table 2:** Thermal properties obtained from DSC analysis of PS and PS-LDH nanocomposites before and after supercritical CO_2_ foaming.

Sample	Before Foaming					After Foaming				
	*T*_g_ (°C)	Onset (°C)	End (°C)	Breadth (°C)	*ΔCp* (J/g·°C)	*T*_g_ (°C)	Onset (°C)	End (°C)	Breadth (°C)	*ΔCp* (J/g·°C)
PS	88.1	85.2	90.9	5.7	0.256	89.3	86.0	92.34	6.4	1.627
LDH1	93.8	90.9	97.0	6.1	0.372	94.6	90.9	98.2	7.3	2.350
LDH3	98.9	96.4	101.6	5.3	0.300	99.9	97.00	102.9	5.9	0.925
LDH5	85.1	81.0	89.0	8	0.236	89.0	85.7	92.3	6.6	1.684

**Table 3:** Properties derived from the microstructures of the nanocomposite samples.

Foam Properties	PS	LDH1	LDH3	LDH5
**Polymer Density, (*ρ*_f_) (g/cm^3^)**	1.01 ± 0.04	0.97 ± 0.02	1.03 ± 0.01	1.08 ± 0.03
**Foam Density, (*ρ*_m_) (g/cm^3^)**	0.08 ± 0.006	0.21 ± 0.05	0.19 ± 0.02	0.21 ± 0.003
**Relative Foam Density, (*ρ_r_*)**	0.08 ± 0.001	0.22 ± 0.002	0.18 ± 0.001	0.19 ± 0.002
**Average Cell Diameter, (*D*) (mm)**	8.6 ± 0.33	2.3 ± 0.06	0.6 ± 0.02	1.7 ± 0.06
**Cell Density, (*N_o_*) (cells/cm^3^)**	4.45 × 10^9^	3.12 × 10^13^	5.21 × 10^15^	1.13 × 10^14^
**Void Fraction, (*V_f_*) (%)**	92.36	78.07	81.85	97.16

**Table 4:** Compressive properties of PS-LDH nanocomposites in comparison to montmorillonite reinforced polystyrene foams.

Samples	Modulus (MPa)	Strength (MPa)	Yield Strength (MPa)
PS	11.25 ± 0.35	0.49 ± 0.049	0.40 ± 0.014
LDH1	41.44 ± 0.57	3 ± 0.02	1.51 ± 0.587
LDH3	38.86 ± 0.7	3.2 ± 0.07	1.07 ± 0.113
LDH5	39.59 ± 0.4	1.7 ± 0.05	1.02 ± 0.037
MLS1	19.84 ± 0.17	0.49 ± 0.017	0.40 ± 0.034

MLS1 data extracted from Ogunsona et al. [15].
